# Diabetes and heart failure associations in women and men: Results from the MORGAM consortium

**DOI:** 10.3389/fcvm.2023.1136764

**Published:** 2023-04-25

**Authors:** Sucharitha Chadalavada, Jaakko Reinikainen, Jonas Andersson, Augusto Di Castelnuovo, Licia Iacoviello, Pekka Jousilahti, Line Lund Kårhus, Allan Linneberg, Stefan Söderberg, Hugh Tunstall-Pedoe, Karim Lekadir, Nay Aung, Magnus T. Jensen, Kari Kuulasmaa, Teemu J. Niiranen, Steffen E. Petersen

**Affiliations:** ^1^ William Harvey Research Institute, NIHR Barts Biomedical Research Centre, Queen Mary University of London, Charterhouse Square, London, United Kingdom; ^2^ Barts Heart Centre, St Bartholomew’s Hospital, Barts Health NHS Trust, West Smithfield, London, United Kingdom; ^3^ Department of Public Health and Welfare, Finnish Institute for Health and Welfare (THL), Helsinki, Finland; ^4^ Department of Public Health and Clinical Medicine, Skellefteå Research Unit, Umeå University, Skellefteå, Sweden; ^5^ Mediterranea Cardiocentro, Naples, Italy; ^6^ Department of Epidemiology and Prevention, IRCCS Neuromed, Pozzilli, Italy; ^7^ Research Center in Epidemiology and Preventive Medicine—EPIMED, Department of Medicine and Surgery, University of Insubria, Varese, Italy; ^8^ Center for Clinical Research and Prevention, Copenhagen University Hospital—Bispebjerg and Frederiksberg, Copenhagen, Denmark; ^9^ Department of Clinical Medicine, Faculty of Health and Medical Sciences, University of Copenhagen, Copenhagen, Denmark; ^10^ Department of Public Health and Clinical Medicine, Umeå University, Umeå, Sweden; ^11^ Cardiovascular Epidemiology Unit, Institute of Cardiovascular Research, University of Dundee, Dundee, United Kingdom; ^12^ Artificial Intelligence in Medicine Lab (BCN-AIM), Departament de Matemàtiques and Informàtica, Universitat de Barcelona, Barcelona, Spain; ^13^ Steno Diabetes Center Copenhagen, Borgmester Ib Juuls Vej 83, Herlev, Denmark; ^14^ Department of Internal Medicine, University of Turku and Turku University Hospital, Turku, Finland; ^15^ Health Data Research UK, London, United Kingdom; ^16^ National Institute for Health and Care Research, London, United Kingdom

**Keywords:** diabetes, heart failure, sex differences, epidemiology, MORGAM

## Abstract

**Background:**

Diabetes and its cardiovascular complications are a growing concern worldwide. Recently, some studies have demonstrated that relative risk of heart failure (HF) is higher in women with type 1 diabetes (T1DM) than in men. This study aims to validate these findings in cohorts representing five countries across Europe.

**Methods:**

This study includes 88,559 (51.8% women) participants, 3,281 (46.3% women) of whom had diabetes at baseline. Survival analysis was performed with the outcomes of interest being death and HF with a follow-up time of 12 years. Sub-group analysis according to sex and type of diabetes was also performed for the HF outcome.

**Results:**

6,460 deaths were recorded, of which 567 were amongst those with diabetes. Additionally, HF was diagnosed in 2,772 individuals (446 with diabetes). A multivariable Cox proportional hazard analysis showed that there was an increased risk of death and HF (hazard ratio (HR) of 1.73 [1.58–1.89] and 2.12 [1.91–2.36], respectively) when comparing those with diabetes and those without. The HR for HF was 6.72 [2.75–16.41] for women with T1DM vs. 5.80 [2.72–12.37] for men with T1DM, but the interaction term for sex differences was insignificant (*p* for interaction 0.45). There was no significant difference in the relative risk of HF between men and women when both types of diabetes were combined (HR 2.22 [1.93–2.54] vs. 1.99 [1.67–2.38] respectively, *p* for interaction 0.80).

**Conclusion:**

Diabetes is associated with increased risks of death and heart failure, and there was no difference in relative risk according to sex.

## Introduction

The impact of diabetes is a global concern with an estimated 500 million people affected worldwide and its prevalence continues to rise (1). The cardiovascular complications of diabetes have the highest impact on mortality and morbidity in those with diabetes (2, 3). Heart failure is the most common cardiovascular complication, which can be asymptomatic initially and often in the absence of macrovascular ischemic disease (4–6).

Observational studies have noted sex differences in cardiovascular outcomes (2, 7, 8). A meta-analysis which included 12 million people demonstrated a relative increase in the risk of heart failure in women with diabetes compared to men (9). This study found a 47% higher relative risk in women with T1DM compared to men and 9% higher in women with T2DM. However, due to the lack of individual-level data, further investigation was not possible to better understand this observation. This was addressed in our recent study, in which a survival analysis was performed on the UK Biobank population consisting of approximately 500,000 participants (10). We also found that the increased relative risk of HF in women was more prominent in T1DM than T2DM (88% increased relative risk in women compared to men with T1DM, 17% in women with T2DM). Therefore, a hypothesis generated is that those with T1DM are more affected by the underlying pathological processes implicated in the increased risk of heart failure in those with diabetes. In addition, it was shown that this increased relative risk in women with diabetes was present even after adjusting for covariates such as age, body mass index, ethnicity, smoking, and alcohol use as well as confounders such as the presence of hypertension, hypercholesterolemia and coronary disease. Competing risk and mediation analysis also supported these findings, which was not possible to discern with the metanalysis.

It is not clear whether the increased relative risk seen for heart failure in women with diabetes, in particular T1DM, is generalizable to other populations. Therefore, we aim to validate the findings from the UK Biobank in external cohorts harmonized in the MORGAM (MOnica Risk, Genetics, Archiving and Monograph) study to better understand the effect of diabetes and sex on the risk of heart failure. This study provides a unique opportunity to assess whether the findings generated from standardized cohorts like the UK Biobank, can be replicated in cohorts representing populations spanning across Europe.

## Materials and methods

### Study cohorts

MORGAM is a multinational study aiming to explore associations of cardiovascular diseases with their classic and genetic risk factors and biomarkers using harmonized data from several population-based cohorts (11). Relevant data for this study were available from five countries: three cohorts from DAN-MONICA Study (Denmark, baseline measurements in 1982–1992), five cohorts from FINRISK Study (Finland, 1982–2002), one cohort from Moli-sani Study (Italy, 2005–2010), six cohorts from Northern Sweden MONICA Study (Sweden, 1986–2009) and four cohorts from Scottish Heart Health Extended Cohort (SHHEC) Study (United Kingdom, 1984–1995).

**Figure 1:**
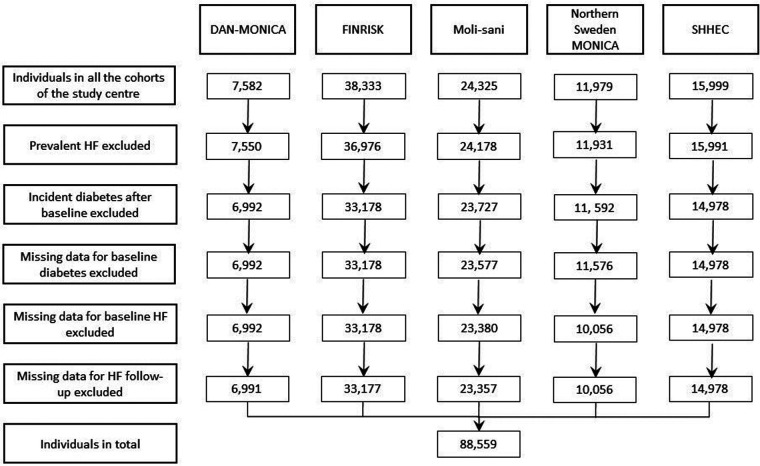
Flowchart of the numbers of participants from different MORGAM centers after data exclusion steps. SHHEC, Scottish Heart Health Extended Cohort; MONICA, Monitoring of Trends and Determinants in Cardiovascular Disease; HF, heart failure.

Figure 1 shows the numbers of participants from the MORGAM Centers after applying various exclusion criteria. After removing individuals with prevalent heart failure (HF) at baseline, incident diabetes after baseline and missing data for baseline diabetes, baseline HF or HF follow-up, the data from 88,559 subjects in total remained. At baseline 3,281 individuals were diabetic (including both type 1 and 2 diabetes) and 85,278 were non-diabetic.

### Study design

As the aim of this study was to externally validate the UK Biobank's findings (10), our analytical approach including variable definitions was made as similar as possible to this previous work. The response variable was the first diagnosis of HF during follow-up. To improve comparability, the follow-up time was restricted to the maximum of 12 years. The number of incident HF cases was 2,772 within this restricted 12-year period.

Prevalent diabetes, including both type 1 and 2, was defined as self-reported or documented diabetes at baseline. Documented history of type 1 diabetes was available only for DAN-MONICA and FINRISK studies and documented history of type 2 diabetes was available for DAN-MONICA, FINRISK and Northern Sweden MONICA studies. Consequently, separate analyses by diabetes type were restricted to DAN-MONICA and FINRISK studies.

The diagnostic criteria for prevalent diseases and follow-up procedures of incident events vary by country and year. Baseline diseases were defined using data from hospital discharge registers, drug reimbursement registers and survey questionnaires. Follow-up data were obtained from causes-of-death registers, hospital discharge registers and death certificates. Further details of disease diagnostics, follow-up procedures and recruitment of each cohort are available online (12).

Baseline coronary disease was defined as documented or self-reported history of myocardial infarction or documented history of cardiac revascularization. Hypertension was defined as systolic blood pressure >140 mmHg, diastolic blood pressure >90 mmHg or use of antihypertensive medication. Hypercholesterolemia was defined as total serum cholesterol ≥6 mmol/L or taking drugs for lowering cholesterol levels.

Smoking history had three categories: current smoker, previous smoker and never smoked. Body mass index (BMI) was derived from measured height and weight as kg/m^2^. The history of alcohol consumption was not as comprehensively available as in survival analysis study performed in UK Biobank population (10), so we used the average daily consumption of alcohol (grams). Information on ethnicity was available only from DAN-MONICA and Moli-sani studies and limited to only two categories (European or other) in MORGAM data, so it was not possible to harmonize the variable to be comparable with the UK Biobank variable (four categories). Thus, we did not include ethnicity in Cox proportional hazard analysis.

### Statistical analyses

The risk of HF against time in those with and without diabetes was visualized by plotting the cumulative probabilities of HF. Associations of diabetes status with heart failure in men and women were assessed by estimating hazard ratios (HR) with Cox proportional hazards models, which were also stratified by cohort. The models were fitted with an interaction effect of sex and diabetes as well as separately for data split by sex. Age, hypertension, smoking, BMI, hypercholesterolemia, alcohol consumption and coronary disease were used as covariates. Further details of the variable definitions and their use in the modelling are described in Supplementary Table S1.

The analyses were carried out both without adjustment for the competing risk of non-HF death and with adjustment using the Fine-Gray model (13). The timescale of the Cox models was the follow-up time which aligned with the UK Biobank analyses.

Missing data were handled using multiple imputation with random forest as the imputation method. The number of imputed datasets was ten. All analyses were carried out using R statistical software, version 4.2.1 (R Core Team) (14). R-package mice (15) was used for the imputation, survival-package (16) for the Cox models and crrSC-package (17) for the competing risks analyses.

### Ethics declarations

The included studies have been approved by local ethic committees as follows: FINRISK Study: 1980s: no ethics approval required for observational studies, but there is a law which allows the use of these data for public health research, 1990s: Ethics committee of the National Public Health Institute (KTL), 2002: Ethics Committee of Epidemiology and Public Health in Hospital District of Helsinki and Uusimaa. DAN-MONICA Study: Ethics Committee of the Capital Region (formerly Copenhagen County), Denmark. Northern Sweden MONICA Study: Research Ethics Committee of Umeå University. Moli-sani Study: Università Cattolica del Sacro Cuore Facoltà di Medicina e Chirurgia “Agostino Gemelli”, Rome. SHHEC Study: Ethical approval was received from all relevant medical research ethics committees covering the individual populations involved.

## Results

The participant characteristics are presented in Table 1. A total of 51.8% of participants were women, but only 46.3% of individuals with diabetes were women. People with diabetes were older, had higher BMI, were more likely to be hypertensive, less likely to be current smokers and had more coronary disease at baseline and more HF events during the follow-up. The amount of missing data was relatively low, except for ethnicity which was used only in a sensitivity analysis. Unadjusted absolute risk of heart failure is higher in men (16.2% vs. 10.5% of women with diabetes experienced HF in the 12 years follow-up period).

**Table 1:** Baseline characteristics and HF follow-up of the participants.

	Overall	Diabetes (men)	No diabetes (men)	Diabetes (women)	No diabetes (women)	Missing, *n* (%)
*N*	88,559	1,763	40,964	1,518	44,314	
*N* by centre (%)
DAN-MONICA	6,991 (7.9)	66 (3.7)	3,413 (8.3)	59 (3.9)	3,453 (7.8)	
FINRISK	33,177 (37.5)	524 (29.7)	15,161 (37.0)	601 (39.6)	16,891 (38.1)	
Moli-sani	23,357 (26.4)	863 (49.0)	10,284 (25.1)	648 (42.7)	11,562 (26.1)	
N. Sweden MONICA	10,056 (11.4)	227 (12.9)	4,709 (11.5)	156 (10.3)	4,964 (11.2)	
SHHEC	14,978 (16.9)	83 (4.7)	7,397 (18.1)	54 (3.6)	7,444 (16.8)	
Baseline age, mean (SD)	49.06 (12.64)	60.41 (11.30)	48.94 (12.55)	56.85 (12.86)	48.46 (12.47)	0 (0.0)
Non-European, *n* (%)	294 (1.0)	5 (0.5)	128 (0.9)	7 (1.0)	154 (1.0)	58,444 (66.0)^a^
Baseline coronary disease, *n* (%)	2,152 (2.4)	216 (12.3)	1,424 (3.5)	73 (4.8)	439 (1.0)	181 (0.2)
Hypertension, *n* (%)	37,268 (42.4)	1,319 (75.2)	18,934 (46.6)	996 (66.0)	16,019 (36.4)	637 (0.7)
Hypercholesterolemia, *n* (%)	36,203 (41.3)	559 (32.1)	17,141 (42.3)	612 (40.8)	17,891 (40.8)	943 (1.1)
BMI, mean (SD)	26.46 (4.50)	29.27 (4.74)	26.62 (3.82)	30.09 (6.32)	26.07 (4.87)	797 (0.9)
Smoking, *n* (%)						494 (0.6)
Current	28,010 (31.8)	444 (25.3)	15,284 (37.5)	235 (15.6)	12,047 (27.4)	
Never	38,346 (43.5)	453 (25.8)	12,856 (31.5)	1,005 (66.8)	24,032 (54.6)	
Previous	21,709 (24.7)	860 (48.9)	12,627 (31.0)	265 (17.6)	7,957 (18.1)	
Alcohol use (g/day), mean (SD)	11.01 (18.18)	17.59 (23.37)	17.54 (22.97)	3.79 (7.86)	4.96 (8.32)	2,213 (2.5)
HF follow-up time, mean (SD)	10.04 (2.60)	7.96 (3.24)	9.98 (2.69)	8.83 (2.92)	10.21 (2.42)	0 (0.0)
HF, *n* (%)	2,772 (3.1)	286 (16.2)	1,341 (3.3)	160 (10.5)	985 (2.2)	0 (0.0)

SHHEC, Scottish Heart Health Extended Cohort; SD, standard deviation; HF, heart failure.

^a^ Missingness of ethnicity led to exclusion as covariate.

**Figure 2:**
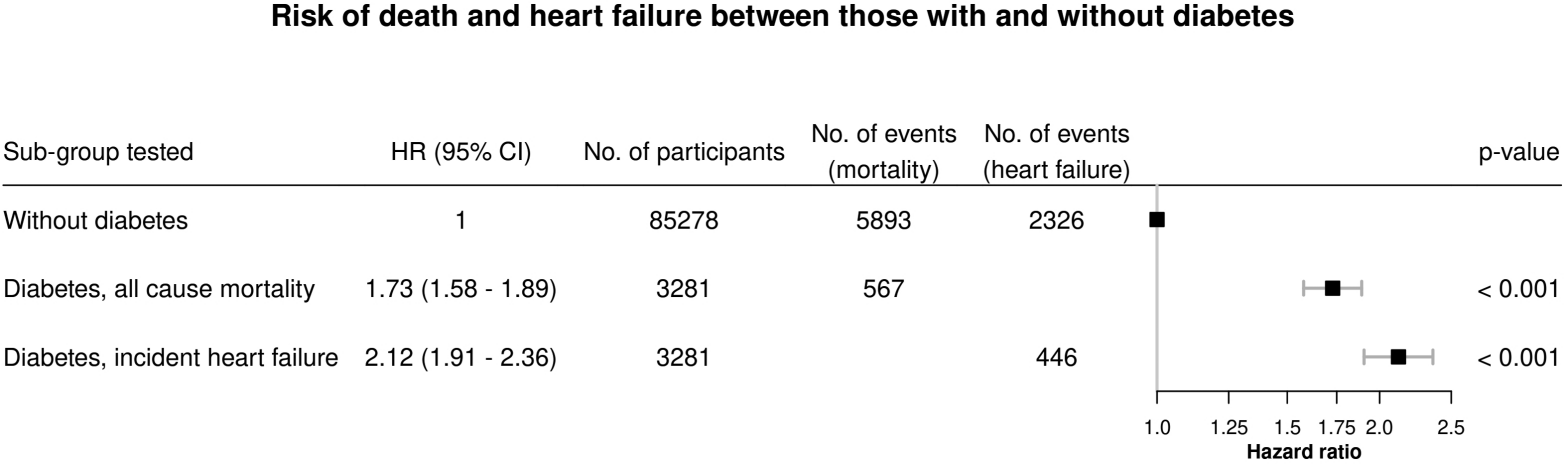
Hazard ratios (HR) with 95% confidence intervals (CI) for diabetes (both types) from separate models with all-cause mortality and heart failure as responses. Adjusted for age, sex, hypertension, hypercholesterolemia, smoking, BMI, alcohol use and coronary artery disease at baseline.

Cumulative incidence of HF was higher in those with diabetes than without diabetes (Supplementary Figure S1). Results from multivariable adjusted hazard ratios (HR) for diabetes (both types) were 1.73 (1.58–1.89) for all-cause mortality and 2.12 (1.91–2.36) for HF (Figure 2).

**Figure 3:**
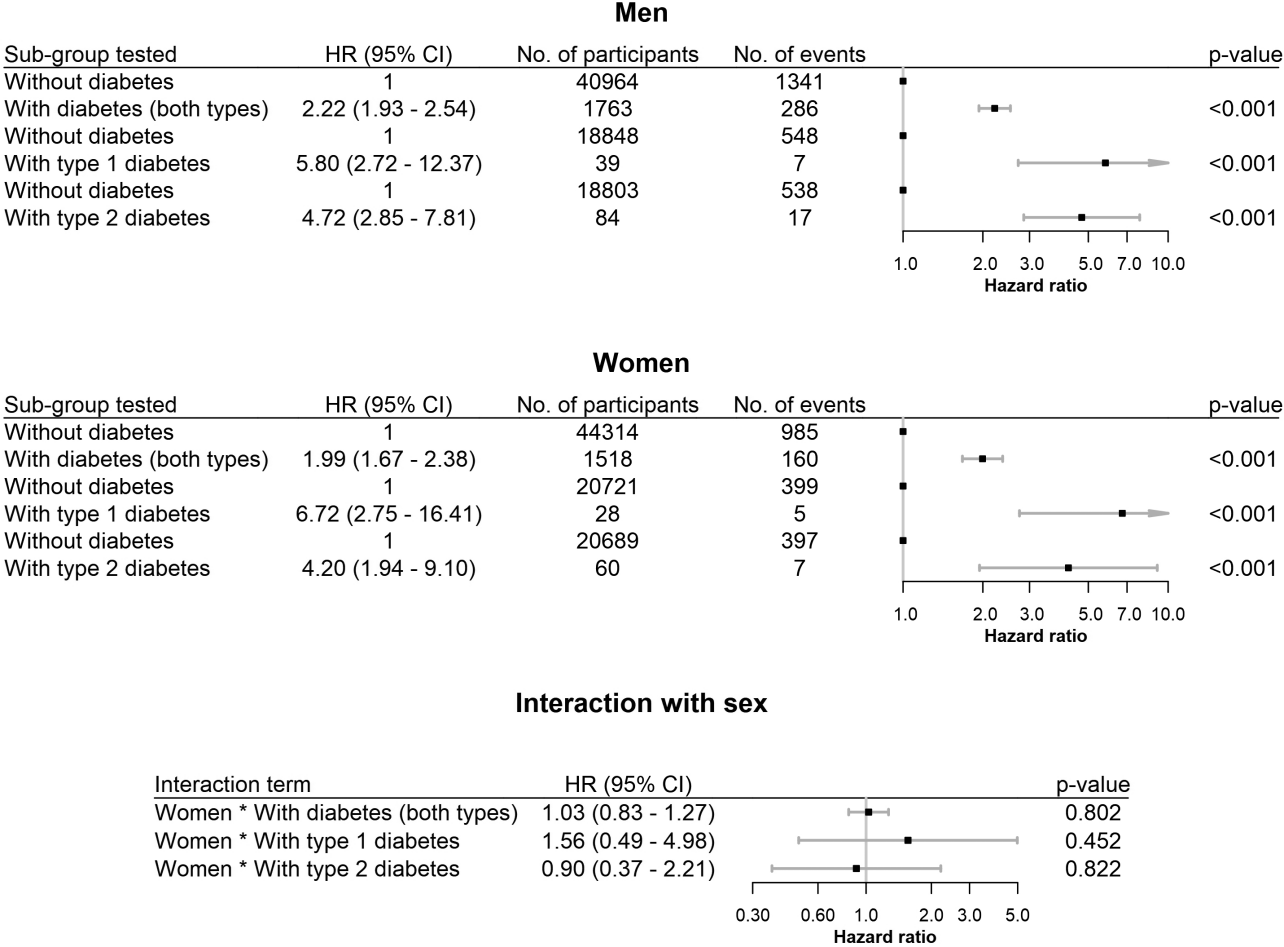
Hazard ratios (HR) with 95% confidence intervals (CI) for diabetes and sex and diabetes interactions on heart failure. Results are from separate models for men, women and both sexes and different types of diabetes adjusted for age, hypertension, hypercholesterolemia, smoking, BMI, alcohol use and coronary artery disease at baseline. Models with both types of diabetes combined use all the cohorts, whereas models with separate variables for type 1 and type 2 diabetes use only cohorts from DAN-MONICA and FINRISK Studies.

Individuals with diabetes had a markedly higher risk of HF than those without diabetes, for both men and women (Figure 3). This relationship was observed regardless of the diabetes type. It should be noted that the estimates for the subtypes of diabetes are not comparable with results for models that include both types of diabetes. This is due to DAN-MONICA and FINRISK are the only studies that have the type of diabetes defined from documentation as opposed to self-reported data source, whereas the other cohorts include self-reported and documented diabetes, but not the sub-type.

The interaction estimates did not demonstrate differences in the associations of diabetes with relative risk of HF between men and women. Due to limited data, the confidence intervals for subtype-specific estimates were very wide. None of the studies included showed any significant difference in relative risk of HF according to sex (see Supplementary Table S2 for further details). Sensitivity analyses using models with adjustment for competing risk of non-HF death resulted in slightly smaller estimates (Supplementary Table S3) but did not change the conclusions.

## Discussion

The results from this study show that the risk of death and heart failure is higher in those with diabetes compared to those without. This confirms the findings seen in the survival analysis performed in the UK Biobank cohort and numerous other epidemiological studies. The focus of this study was to better understand the impact of sex on the outcome of heart failure for people with diabetes.

The results which included all the MORGAM cohorts which fit the inclusion criteria of this paper, showed, as expected, that the absolute risk of heart failure is higher in men. The increased absolute risk of cardiovascular outcomes being higher in men (regardless of diabetes status) has been well documented (18–20). This study was focusing on the increased relative risk of heart failure in women with diabetes compared to men as demonstrated in other studies (9, 10), which suggests that the protection from adverse cardiovascular outcomes offered by the female sex, is attenuated in those with diabetes (21). The results in this study did not show any sex-specific differences in the relative risk of heart failure when comparing men and women with diabetes (both types) with their non-diabetic counterparts (HR of 2.22 vs. 1.99 respectively). This deviates from the findings in the UK Biobank study as well as a large meta-analysis which did report an increased relative risk of heart failure in women with diabetes compared to men with diabetes (9, 10).

There are several reasons that could explain our contrasting and negative findings. There is the possibility of missing an existing effect in our population due to insufficient sample size and power of men and women with type 1 diabetes. It is type 1 diabetes that seems to drive the higher relative risk of heart failure in women in the literature rather than type 2 diabetes. In the UK Biobank cohort, the interaction of sex and diabetes was the strongest and statistically most significant with type 1 diabetes (T1DM), whereas the interaction term was insignificant for type 2 diabetes (10). Findings in the meta-analysis which included 12 million people also reflected this trend, where those T1DM were affected more than those with T2DM, but did not have interaction term analysis to determine statistical significance based on type of diabetes, due to lack of individual level data (9). Our analysis performed in the two MORGAM cohorts with information on type of diabetes (DAN-MONICA and FINRISK) indicated a trend towards an increased relative risk of heart failure in women with T1DM (HR of 6.72 in women with T1DM vs. 5.80 in men with diabetes) despite the interaction term being insignificant. However, our findings may also be negative for reasons other than reduced power. It is possible that there are disparities related to sex in the detection of risk factors such as diabetes and outcomes such as heart failure across different countries and healthcare systems included in the MORGAM consortium, which may partly explain the negative findings in this study. Additionally, many of the studies in the MORGAM consortium derive their data from as early as 1980s when the diagnosis of conditions such as diabetes (including sub-types) and heart failure were not as well established as they are in contemporary studies. The UK Biobank differs from the data in this study as the UK Biobank is comprised of more recent data collected prospectively within a single country with a more standardized healthcare provider. Similarly, the metanalysis of 12 million people may reflect epidemiological association between sex and heart failure, which are not seen in more heterogenous and historic populations like those included in this study.

One reason for why those with T1DM are possibly affected more may be due to the duration of diabetes, which would typically be longer than those with T2DM. Prolonged period of exposure to hyperglycemia could activate and sustain the inflammatory pathways implicated in an altered metabolism which could lead to adverse cardiac remodeling known as diabetic cardiomyopathy (22, 23). A recent study has demonstrated that a deterioration in strain measurements (E/e′ and GLS), which are thought to be a hallmark of diabetic cardiomyopathy are associated with major adverse cardiovascular events (MACE) in women but not in men (24). Hyperinsulinemia has also been implicated as a contributor to adverse cardiac modeling (22, 25, 26) which could explain the differences in observed cardiovascular consequences between those with T1DM and T2DM.

Further studies need to be performed which distinguishes not only by the type of diabetes, but also glycemic control, insulin treatment and the duration of diabetes. These studies could provide further evidence to support or refute the hypothesis that those with T1DM, in particular women, are disproportionately affected by the processes that lead to an increased risk of heart failure in diabetes.

### Strengths and limitations

A major strength of our study is the multicenter, multi-country, individual-level harmonized data. One of the limitations of this validation study is that despite the overall large sample there were only 3,281 participants with diabetes compared to 22,300 in the UK Biobank study. The smaller sample size also didn't allow for mediation analysis to be performed to further assess to what extent risk factors such as coronary disease are mediating the increased risk of heart failure, which would further inform potential underlying mechanisms. In particular, there were only 67 participants with T1DM in this study, which is the principal sub-group of interest, compared to 2,626 participants with type 1 diabetes in the UK Biobank study. This reflects the historic nature of the data represented in this study where some studies were established when the detailed sub-typing of diseases was not a standard practice.

Another limitation is the lack of information on ethnicity. This is a majority white ethnicity population as was the case with the UK Biobank study. It is possible that other ethnicities may be more sensitive to the cardiovascular changes caused by diabetes, but this cannot be studied due to the lack of participants from other ethnicities, which affects the applicability of the findings to the wider world population. On the other hand, the MORGAM study includes data from several countries with different healthcare systems, therefore is perhaps a more representative cohort of the general white population.

## Conclusion

A survival analysis performed on harmonized cohorts in the MORGAM study demonstrated that those with diabetes have a significantly higher risk of death and heart failure compared to those without. This is in keeping with the survival analysis performed in UK Biobank and many other epidemiological studies. However, overall, this study was not able to demonstrate the difference in relative risk of heart failure based on sex in those with diabetes. A smaller sub-study which distinguished participants by the type of diabetes suggested that women with T1DM may have a higher relative risk of heart failure, but this difference was not statistically significant. These findings have added support to the theory that the increased relative risk of heart failure seen in women with diabetes in the larger studies may be mostly driven due to the inclusion of larger numbers of participants with T1DM who are possibly disproportionately affected.

## Data Availability

The data analyzed in this study is subject to the following licenses/restrictions: The MORGAM data is not available in a public repository. Access to the data is restricted by the ethical approvals and the legislation of the European Union and the countries of each study. Approval by the Principal Investigator of each cohort study and the MORGAM/BiomarCaRE Steering Group will be required for release of the data. The MORGAM Manual at https://www.thl.fi/publications/morgam/manual/contents.htm gives more information on access. Requests to access these datasets should be directed to https://www.thl.fi/publications/morgam/manual/contents.htm.
