# Supplementary material for: Diabetes and heart failure associations in women and men: Results from the MORGAM consortium
Source: Front Cardiovasc Med. 2023 Apr 25;10:1136764. doi: 10.3389/fcvm.2023.1136764 (PMC10167048; doi:10.3389/fcvm.2023.1136764)
Supplement: Supplementary file 1 [file Datasheet1.pdf]

Supplementary material

Supplementary Table 1. Definitions and types of variables used in the modelling.

Variable	Definition	Type in modelling
Diabetes (both types)	Self-reported or documented diabetes at baseline	Factor with two categories
Type 1 diabetes	Documented history of type 1 diabetes	Factor with two categories
Type 2 diabetes	Documented history of type 2 diabetes	Factor with two categories
Sex	Man or woman	Factor with two categories
Age	Age at baseline in years	Continuous
Hypertension	Systolic blood pressure > 140 mmHg, diastolic blood pressure > 90 or use of antihypertensive medication	Factor with two categories
Hypercholesterolemia	Total serum cholesterol ≥ 6 mmol/l or taking drugs for lowering cholesterol levels. Information on the cholesterol medication was used only from the year 1999 onwards	Factor with two categories
Smoking	Current smoker, previous smoker or never smoked	Factor with three categories
Body mass index (BMI)	Derived from measured height and weight as kg/m^2	Continuous
Alcohol use	Average daily consumption of alcohol in grams	Continuous with a non-linear effect. Modelled by using restricted cubic splines[a]. Based on a graphical inspection, two inner knots were set at 3 and 6 grams to capture the shape of the relationship between alcohol consumption and the response
Coronary artery disease	Documented or self-reported history of myocardial infarction or documented history of cardiac revascularization	Factor with two categories
Ethnicity	European or other	Factor with two categories
Heart failure	The first HF diagnosis during follow-up	Time-to-event variable, right-censored
Death	Death from any cause during follow-up	Time-to-event variable, right-censored

[a] Marrie RA, Dawson NV, Garland A. Quantile regression and restricted cubic splines are useful for exploring relationships between continuous variables. J Clin Epidemiol. 2009; 62(5):511-517.

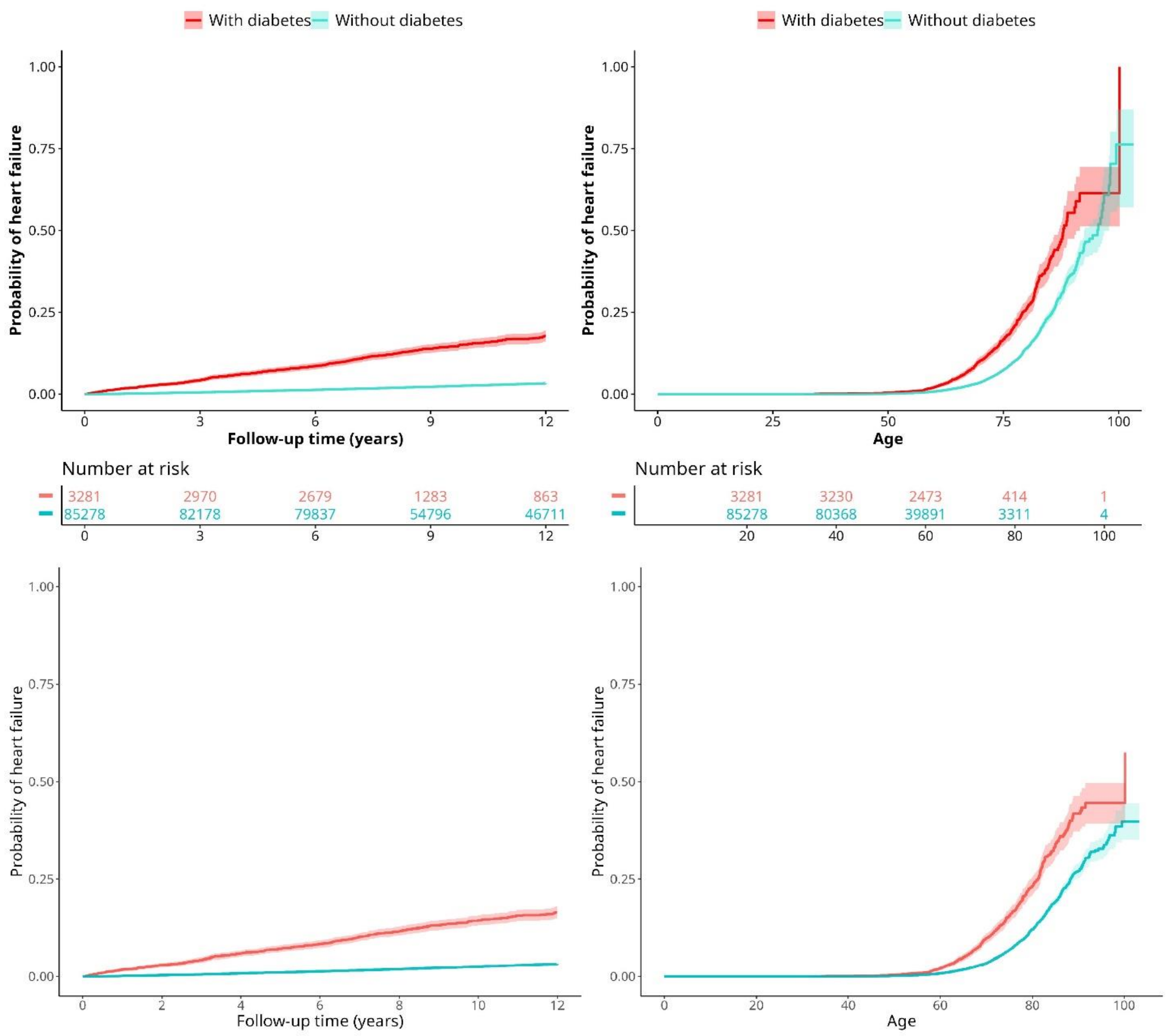

Supplementary figure 1. Cumulative probability of heart failure in those with and without diabetes. The upper panels are without and lower with adjustment for competing risk of non-HF death. The left-hand side panels are from the analyses with follow-up time as the time-scale and right-hand side panels with age as the time-scale.

*Supplementary Table 2. Hazard ratios (HR) with 95% confidence intervals (CI) for diabetes (including both types) and sex*diabetes interactions on heart failure separately for the five MORGAM Centers. Results are from models separately for men, women and both sexes. Models are adjusted for age, hypertension, hypercholesterolemia, smoking, BMI, alcohol use and coronary artery disease at baseline.*

MORGAM Center	Sex	Term	HR (95% CI)	No. of participants	No. of events
DAN-MONICA	Men	Without diabetes	1	3,413	104
		With diabetes	3.72 (2.02–6.85)	66	14
	Women	Without diabetes	1	3,453	67

		With diabetes	2.55 (0.96–6.81)	59	5
	Interaction	Women * With diabetes	0.65 (0.22–1.93)		
FINRISK	Men	Without diabetes	1	15,161	415
		With diabetes	2.28 (1.76–2.96)	524	74
	Women	Without diabetes	1	16,891	315
		With diabetes	1.83 (1.34–2.49)	601	49
	Interaction	Women * With diabetes	0.93 (0.63–1.38)		
SHHEC	Men	Without diabetes	1	7,397	242
		With diabetes	3.01 (1.67–5.43)	83	13
	Women	Without diabetes	1	7,444	136
		With diabetes	2.04 (0.72–5.76)	54	4
	Interaction	Women * With diabetes	0.88 (0.28–2.80)		
Northern Sweden	Men	Without diabetes	1	4,709	105
		With diabetes	3.07 (1.99–4.71)	227	33
	Women	Without diabetes	1	4,964	75
		With diabetes	3.72 (2.22–6.25)	156	20
	Interaction	Women * With diabetes	1.24 (0.66–2.36)		
Moli-sani	Men	Without diabetes	1	10,284	475
		With diabetes	2.01 (1.65–2.43)	863	152
	Women	Without diabetes	1	11,562	392
		With diabetes	2.00 (1.56–2.57)	648	82
	Interaction	Women * With diabetes	1.01 (0.75–1.36)		

*Supplementary Table 3. Subdistribution hazard ratios (sHR) with 95% confidence intervals (CI) for diabetes and sex*diabetes interactions on heart failure. Results are from models adjusted for competing risk of non-HF death separately for men, women and both sexes and different types of diabetes. Models are adjusted for age, hypertension, hypercholesterolemia, smoking, BMI, alcohol use and coronary artery disease at baseline. Reference groups are those without diabetes. Models with both types of diabetes combined use all*

the cohorts, whereas models with separate variables for type 1 and type 2 diabetes use only cohorts from DAN-MONICA and FINRISK Studies.

	**Term**	**sHR (95% CI)**
Men	With diabetes (both types)	2.11 (1.82–2.45)
	With type 1 diabetes	6.06 (2.95–12.41)
	With type 2 diabetes	4.58 (2.76–7.59)
Women	With diabetes (both types)	1.92 (1.60–2.30)
	With type 1 diabetes	6.45 (2.44–17.03)
	With type 2 diabetes	3.59 (1.67–7.72)
Interaction with sex	Women * With diabetes (both types)	1.02 (0.82–1.28)
	Women * With type 1 diabetes	1.41 (0.42–4.71)
	Women * With type 2 diabetes	0.81 (0.33–2.00)
